# *Bacillus vallismortis* TU-Orga21 blocks rice blast through both direct effect and stimulation of plant defense

**DOI:** 10.3389/fpls.2023.1103487

**Published:** 2023-02-20

**Authors:** Wannaporn Thepbandit, Anake Srisuwan, Supatcharee Siriwong, Siriwan Nawong, Dusit Athinuwat

**Affiliations:** ^1^Faculty of Science and Technology, Thammasat University, Pathumtani, Thailand; ^2^Faculty of Science and Technology, Nakhon Ratchasima Rajabhat University, Nakhon Ratchasima, Thailand; ^3^Synchrotron Light Research Institute, Nakhon Ratchasima, Thailand; ^4^Center of Excellence in Agriculture Innovation Centre through Supply Chain and Value Chain, Thammasat University, Pathumtani, Thailand

**Keywords:** plant health, lipopeptide biosurfactant, biotic stress, phytohormone, plant-immunity, sustainable agriculture, plant–microbe interaction, rice disease

## Abstract

Beneficial microorganisms are an important strategy for sustainable plant production processes such as stimulate root exudation, stress tolerance, and yield improvement. This study investigated various microorganisms isolated from the rhizosphere of *Oryza sativa* L. in order to inhibit *Magnaporthe oryzae* cause of rice blast, by direct and indirect mode of action. The results indicated that *Bacillus vallismortis* strain TU–Orga21 significantly reduced *M. oryzae* mycelium growth and deformed the hyphal structures. The effects of biosurfactant TU–Orga21 was studied against *M. oryzae* spore development. The dose of ≥5% *v*/*v* biosurfactant significantly inhibited the germ tubes and appressoria formation. The biosurfactants were evaluated as surfactin and iturin A by Matrix-assisted laser desorption ionization dual time-of-flight tandem mass spectrometry. Under greenhouse conditions, priming the biosurfactant three times before *M. oryzae* infection significantly accumulated endogenous salicylic acid, phenolic compounds, and hydrogen peroxide (H_2_O_2_) during the infection process of *M. oryzae*. The SR-FT-IR spectral changes from the mesophyll revealed higher integral area groups of lipids, pectins, and proteins amide I and amide II in the elicitation sample. Furthermore, scanning electron microscope revealed appressorium and hyphal enlargement in un-elicitation leaves whereas appressorium formation and hyphal invasion were not found in biosurfactant-elicitation at 24 h post inoculation. The biosurfactant treatment significantly mitigated rice blast disease severity. Therefore, *B. vallismortis* can be a promising novel biocontrol agent which contains the preformed active metabolites for a rapid control of rice blast by a direct action against pathogen and by boosting plant immunity.

## 1 Introduction

Rice (*Oryza sativa* L.) production is negatively affected by various factors, such as poor soil quality, mineral deficiency, pests, and diseases, among others, ultimately reducing rice yield (Timsina et al., 2018). One of the major diseases of rice is blast caused by *Magnaporthe oryzae* (Howard and Valent, 1996). The rice blast disease is widespread across Southeast Asia and Japan and is responsible for yield losses of 20%–50% on average and even up to 100% in severe cases (Saleh et al., 2014). In Thailand, rice blast is a serious threat to rice production systems because of favorable climatic conditions to disease spread (Kawasaki and Herath, 2018). Therefore, reducing the prevalence and severity of rice blast is a priority for improving the yield and reducing the losses in Thai rice production. At present, the major control methods for rice blast rely on the application of synthetic chemicals, such as captan, carbendazim, thiram, or tricyclazole (Kongcharoen et al., 2020). However, these components may negatively affect humans and the environment. Therefore, novel bioproducts must be explored to control rice blast.

Currently, biocontrol of rice diseases using microorganisms, biomass, or crude extracts of microorganisms is an exciting new avenue in the development of biofungicides, which are safe for humans, animals, and the environment. These agents hold the potential to control plant diseases through promoting antibiotic production, competition, parasitism, and plant health (Köhl et al., 2019; Khanna et al., 2019). In addition, synthesizing various antimicrobial agents or toxins in order to directly attack the pathogens also activates the systemic resistance mechanisms of plants and enhances the defense enzyme properties (Khanna et al., 2021; Abdelaziz et al., 2023).

*Bacillus* species are potential candidates for developing biofungicides as well as a method to combat bacterial rice pathogens since they produce multifarious active compounds and strong antimicrobial activity such as lipopeptides antibiotics (Hashizume and Nishimura, 2008; Sharma and Thakur, 2020; Ngalimat et al., 2021). Lipopeptides are a group of antimicrobial metabolites composed of surfactin, lichenycin, iturin, and fengycin. These substances affect cellular integrity and can destroy the fungal membrane (Romero et al., 2007). According to Zhang and Sun (2018), cyclic lipopeptides and fengycin produced by *Bacillus subtilis* 155 can damage rice membrane, inhibiting *M. grisea* hyphal growth (Zhang and Sun, 2018). Similarly, *B. amyloliquefaciens* S170 and *B. pumilus* S9 can colonize rice plants to protect them against *M. oryzae* by producing proteases, siderophores, cellulases, and volatile compounds (Sha et al., 2020).

Moreover, lipopeptide substances secreted by *Bacillus* sp. are involved in the elicitation of induced systemic resistance (ISR) to inhibit a wide range of plant pathogens such as fungi, bacteria, nematodes, insects, and viral vectors (Jourdan et al., 2009; Malviya et al., 2020). ISR is a systemic means of inducing preconditioned resistance through prior infection or biotic treatment. Preconditioning induces resistance, rendering the host less susceptible to subsequent infection and blocking the spread of infection in plant tissues (Romera et al., 2019). In the mechanism of ISR, small molecules and cellular tissues in the plant cell wall structure are modified, and antimicrobial compounds are synthesized through the salicylic acid (SA) pathway (Heil and Bostock, 2002; Deice Raasch-Fernandes et al., 2019). Following infection, endogenous levels of SA increase locally, thereby systemically enhancing plant defense mechanisms through the shikimic acid pathway and producing phenolic compounds in plant cell walls and lignin in response to a pathogen attack (Mandal et al., 2010). Moreover, according to Sedlářová and Lebeda (2001), rapid accumulation of phenolic compounds was associated with a hypersensitive response (HR) to downy mildew in lettuce (Sedlářová and Lebeda, 2001). Furthermore, in groundnut, plant growth-promoting rhizobacterial (PGPR) bioformulation enhanced the expression of defense enzymes and pathogenesis-related proteins against collar rot *via* augmenting the enzymatic activity of phenolics (Senthilraja et al., 2013)

Although beneficial microbes, such as *Bacillus* species, have been proven to effectively suppress *M. oryzae*, the biocontrol potential of their multiple modes of action against pathogens has not been fully exploited as yet. Therefore, the objective of the present study was to explore the potential benefits of microbes for the inhibition of *M. oryzae*, their antimicrobial compounds, and their direct inhibiting potential on *M. oryzae* spore development in parallel with stimulation of the plant defense intermedia including endogenous salicylic acid, phenolic compound, and H_2_O_2_ accumulation. Moreover, we also analyzed the biochemical changes including lipid, pectin, amides I, amides II, lignin, and polysaccharides in rice plants during *M. oryzae* infection.

## 2 Materials and methods

### 2.1 Microorganisms, cell culture conditions, and cell-free biosurfactant

One gram of soil sample in the rhizospheric region of rice plants was diluted into 9 mL of distilled water to obtain a serially diluted suspension at 10^-8^ g mL^-1^ for assays. Thereafter, 100 µL of the soil suspension was dropped on nutrient agar (NA) plates and spread over the surface of the agar with a sterile glass spreader; the plates were then incubated at 28°C for 48 h. A single colony of microorganisms was selected as the pure strain through the streak plate method for matrix-assisted laser desorption ionization dual time-of-flight tandem mass spectrometric (MALDI-TOF/TOF MS) identification and further experimentation.

To prepare the biosurfactant, the microorganisms were cultured in flasks containing 100 mL of lysogeny broth (LB) at 25°C ± 2°C and 180 rpm for 72 h. The culture was centrifuged (12,000 ×*g*, 25 min) to produce the biosurfactant without the cell biomass. The biosurfactant was flowed through a membrane filter with 0.45 µm pore size to remove the cellular residue and then preserved at 4°C for further experimentation.

Biosurfactant lipopeptides were precipitated by adding hydrochloric acid (3N) solution; the mixture was cooled down to 4°C to obtain the final pH of 2.0 and then placed in a fridge for 1 h. The precipitates were collected by centrifugation at 9,000 ×*g* for 25 min at 4°C and then dissolved in chloroform:methanol (2:1, *v*/*v*) before MALDI–TOF/TOF MS analysis.

A virulent strain of *M. oryzae* was provided by the Plant Pathology Section of Thammasat University. *M. oryzae* was grown on oatmeal–rice agar medium at 25°C ± 2°C for 10 days. The conidia were harvested using a loop *via* gentle scraping. The suspensions were filtered through layers of cheesecloth to obtain pure conidia, and their density was adjusted to approximately 1 × 10^5^ conidia mL^-1^ using a hemocytometer.

### 2.2 Rice cultivar and planting

Thai jasmine rice cultivar ‘Khao Dowk Mali 105’ (KDML 105), which is susceptible to blast disease, was used in the present experiment. Rice seeds were sterilized with 1% sodium hypochlorite solution for 1 min and then rinsed with sterile water three times. The seeds were soaked in water 24 h before planting. Ten seedlings were planted in 10–inch plant pots containing approximately 5 kg of clay soil. The pots were incubated in a greenhouse under 12 h of natural light at 28°C ± 4°C with 60%–75% humidity.

### 2.3 Strains identification using MALDI–TOF/TOF MS

MALDI–TOF/TOF MS was performed at the Synchrotron Light Research Institute, Thailand, using Autoflex^®^ maX (Bruker Daltonics, Bremen, Germany). Four to five pure single colonies (5–10 mg) of each microbial strain on NA medium were transferred to 1.5 mL microcentrifuge tubes and then mixed with 300 μL of HPLC–grade water. Next, 900 μL of ethanol was added, and the mixture was vortexed for 1 min. The sample solution was centrifuged at 13,500 ×*g* for 2 min two times. The cells were dried in a laminar air flow cabinet for 2 min, and then 5 µL of 70% formic acid was added. The sample was vortexed for 1 min. Afterwards, 5 µL of acetonitrile was added, and the solution was centrifuged at 13,000 ×*g* for 2 min. At this point, the clear solution was transferred to a fresh tube. Next, 1 μL of each sample was dropped on the MALDI–TOF/TOF MS target plate and left to dry; subsequently, 1 µL of HCCA matrix was dropped and left to dry. The target plates containing the samples were analyzed using MALDI–TOF/TOF MS (Autoflex^®^ maX, Bruker Daltonics, Karlsruhe, Germany). The spectra were recorded in the positive linear mode at the laser frequency of 60 Hz and in the mass range of *m*/*z* 2,000–10,000 Dalton. Calibration was performed using the *Escherichia coli* strain DH5α, which presents ribosomal protein mass with RNA and myoglobin at peaks of 5096.8, 5381.4, 6255.4, 7274.5, 10,300.1, 13,683.2, and 16,952.3 *m*/*z*. Two–thousand shots of the mass spectrum profile data were collected from each sample. The mass spectrum profiles were processed with Flexcontrol v.3.4 (Karlsruhe, Germany). The protein mass fingerprints were analyzed at a molecular weight tolerance of 300 ppm. The resulting data were imported to MALDI BioTyper v.4.0 (Karlsruhe, Germany) to compare the sample mass fingerprints to the database (Elshafie et al., 2015).

### 2.4 *In-vitro* inhibition of mycelial growth

Dual culture technique was used to determine the antagonistic activity of bacterial isolates against *M. oryzae*. *M. oryzae* fungal disks (8 mm in diameter) were placed at the center of 90 mm PDA plates, and five strains of beneficial microorganisms grown in Luria Bertani (LB) broth were streaked at the side of each plate using the dual culture method. LB broth media without the beneficial microorganism was used as the untreated control. The plates were incubated at 25°C ± 2°C. The best bacterial isolate for inhibition of mycelial growth was used for further analysis. Antagonistic activity was calculated based on the observation of mycelial growth at 10 days using formula (1), as follows:


(1) $$\frac{\text{Mycelial growth on control }-\text{Mycelial growth of treated sample}}{\text{Mycelial growth on control}}\;\times 100$$

### 2.5 *B. vallismortis* biosurfactant on suppressed *M. oryzae* spore germination

Leaf segments the susceptible rice cultivar KDML 105 were used for evaluation. Briefly, 5–cm–long samples from the middle part of the leaves were extracted, placed on water agar (WA) plates, and sprayed with 1 × 10^5^ conidia mL^-1^ suspension of *M. oryzae*. After 1 h of inoculation, the rice leaves were sprayed with the *B. vallismortis* biosurfactant at various concentrations (5%, 10%, 15%, 20%, 25%, and 30% *v*/*v*). LB broth without the biosurfactant was used as the negative control. All plates were incubated at 25°C ± 2°C in a growth chamber for 16 h. The leaf segments were stained with ethanol–lactophenol trypan blue (Koch and Slusarenko, 1990). Spore development and appressoria formation were monitored by microscopy. The percentage of spores, germ tubes, and appressorium and the percentage of inhibition of *M. oryzae* spore germination were calculated using formula (2) and (3) respectively as follows:


(2) $$\frac{\text{Number of spores on each stage}}{\text{Total number of spores}}\;\times 100$$

(3) $$\frac{Germinated\;spores\;in\;control\;sample-Germinated\;spores\;in\;treated\;sample\;}{germinated\;spores\;in\;control\;sample}\;\times \;100$$

### 2.6 Characterization of *B. vallismortis* biosurfactant by MALDI–TOF/TOF MS

The lipopeptide biosurfactant was analyzed at the Synchrotron Light Research Institute, Thailand, with Autoflex^®^ maX (Bruker Daltonics, Bremen, Germany) in the positive reflectron mode and the *m*/*z* range of 50–2,000 Dalton. The lipopeptide biosurfactant was mixed with an equal volume of matrix solution (30:70 *v*/*v* ACN : TFA 0.1% TFA), and then 1 μL of the lipopeptide sample was dropped on the plate and left to dry. The standard purified iturin A and surfactin (Sigma-Aldrich, USA) was used as a marker. The spectra were obtained using FlexControl v3.4 (Karlsruhe, Germany) and analyzed using FlexAnalysis v3.3 (Karlsruhe, Germany) (Elshafie et al., 2015).

### 2.7 Potential of *B. vallismortis* biosurfactant to activated plant defense response

#### 2.7.1 Endogenous SA contents

The efficacy of the *B. vallismortis* biosurfactant as the sole regulator to induce systemic resistance was determined at concentrations of 20%, 25%, and 30% *v*/*v*. The evaluated treatments also included SA as the positive control and water as the negative control. Plants were sprayed with 30 mL of the biosurfactant solution at various concentrations and the corresponding control solutions once a week for 3 weeks from day 30 post–sowing. The rice plants were inoculated by spraying 1 × 10^5^ conidia mL^-1^ suspension of *M. oryzae* and then covered with plastic bags for 12 h. Rice leaf samples (0.5 g) were collected from each treatment at 0, 12, 24, 36, 48, and 60 h post–inoculation (hpi) to examine endogenous SA. The leaf samples were soaked in liquid nitrogen (LN2) and homogenized with 1 mL of buffer solution containing 90% methanol, 9% glacial acetic acid, and 1% water by volume unit. The ground samples were centrifuged at 14,000 ×*g* for 10 min at 4°C. Next, 0.5 mL of the supernatant was transferred into an Eppendorf tube containing 0.5 mL of 0.02 M ferric ammonium sulfate, and the sample was incubated for 5 min at 30°C. Subsequently, 200 μL of each sample was transferred to a 96-well plate, and absorbance was measured at 530 nm using a Bio–Tek microplate reader (Winooski, VT, USA). Endogenous SA was calculated through comparison with the standard references (Udayashankar et al., 2011).

#### 2.7.2 Total phenolics compound

The total phenolic content of the leaves was determined following a slightly modified Folin–Ciocalteu assay (Lamuela-Raventós, 2018). Leaf samples (0.5 g) were soaked in LN2 and then ground with 2.5 mL of 80% methanol. The finely ground samples were centrifuged at 14,000 ×*g* for 10 min at 4°C. Next, 0.2 mL of the supernatant was transferred to an Eppendorf tube containing 0.2 mL of Folin–Ciocalteu reagent and mixed by vortexing. Then, 2 mL of 2% sodium carbonate was added prior to incubation for 20 min at 30°C in the dark. Subsequently, 200 μL of each sample was transferred to a 96-well plate, and absorbance was measured at 760 nm using a Bio–Tek microplate reader (Winooski, VT, USA). Gallic acid was used as the calibration standard, and the total phenolic content was expressed as microgram of gallic acid equivalent (g^-1^ FW) on a fresh weight basis (µg GAE g^-1^ FW).

#### 2.7.3 Hydrogen peroxide (H_2_O_2_) accumulation

Rice leaf samples from each treatment (0.5 g) were soaked in LN2, homogenized with 5 mL of 0.1% trichloroacetic acid, and centrifuged at 14,000 ×*g* and 4°C for 15 min. The samples (0.5 mL) were mixed with 0.5 mL of 10 mM potassium phosphate buffer (pH 7.0) and 1 mL of 1 M potassium iodide. Absorbance was measured at 390 nm using a Bio–Tek microplate reader (Winooski, VT, USA) (Sahebani and Hadavi, 2009).

### 2.8 Monitoring rice mesophyll biochemical changes using synchrotron radiation–based–Fourier–transform infrared microspectroscopy

Treated and untreated leaf samples were collected, fixed in the optimal cutting temperature compound, and frozen in LN2. The samples were transversely sectioned at 8 μm using a cryostat microtome (Wetzlar, Germany) and then laid on 13 × 2 mm slides. Spectral derivatives were generated using Beamline 4.1 at the Synchrotron Light Research Institute, Thailand. Measurements were performed by mapping at an aperture size of 10 × 10 μm and spectral resolution mode of 4 cm^-1^ with 64 scans. Spectral derivatives were generated using OPUS 7.2 (Hanau, Germany), The data and principal component analysis (PCA) were analyzed using Cytospec™ and UnscramblerX 10.0 (New Jersey, USA) (Thepbandit et al., 2021).

### 2.9 *M. oryzae* infection process on elicitation-plant

The *M. oryzae* infection process during plant defense response was analyzed using scanning electron microscopy (SEM). Rice plants were spayed for each elicitor three times and then inoculated with *M. oryzae* using 30 mL of the conidial suspension (1 × 10^5^ conidia mL^-1^) per pot, as described above. Thereafter, 5–cm–long leaf samples were collected from different sets of treatments and soaked in phenol to stop the infection process. Finally, the samples were subjected to SEM.

### 2.10 Rice blast disease severity rating

Disease symptoms appearing on the tested rice leaves at 14 days post–inoculation (dpi) were analyzed based on the IRRI standard for rice blast disease scoring (IRRI, 2013), specified as follows:

**Table:** 

Blast disease scale	Plant infection
0	No symptoms
1	Tiny brown spots of a pinhead size
2	Big brown spot
3	Oval necrotic gray speckles of 1–2 mm in diameter with a surrounding brown edge
4	Elliptical necrotic gray spot of 1–2 cm corresponding to symptoms found on<2% of the leaf area
5	Symptoms on<10% of the leaf area
6	Symptoms on 10%–25% of the leaf area
7	Symptoms on 26%–50% of the leaf area
8	Symptoms on 51%–75% of the leaf area
9	Whole leaf is dead

The disease severity index and control efficacy were calculated using the formulas (4) and (5), respectively, as follows:


(4) $$\frac{\text{Σ}\left(\text{Rice blast rating }\times \text{Number of leaves of this rating}\right)}{\text{Total rice leaves }\times \text{maximum rating scale}}\;\times 100$$

(5) $$\frac{\text{Control treatment}-\text{Treated treatment}}{\text{Control treatment}}$$

### 2.11 Statistical analysis

To ensure data consistency, the experiments were performed under greenhouse conditions and repeated three times. The obtained experimental data were analyzed using one–way ANOVA with SPSS 20 (SPSS Inc., Chicago, USA). Significant differences in means among groups were analyzed using Duncan’s multiple range test at *p* ≤ 0.05.

## 3 Results

### 3.1 Identification of *B. vallismortis* TU–Orga21 using MALDI–TOF MS

**Figure 1:**
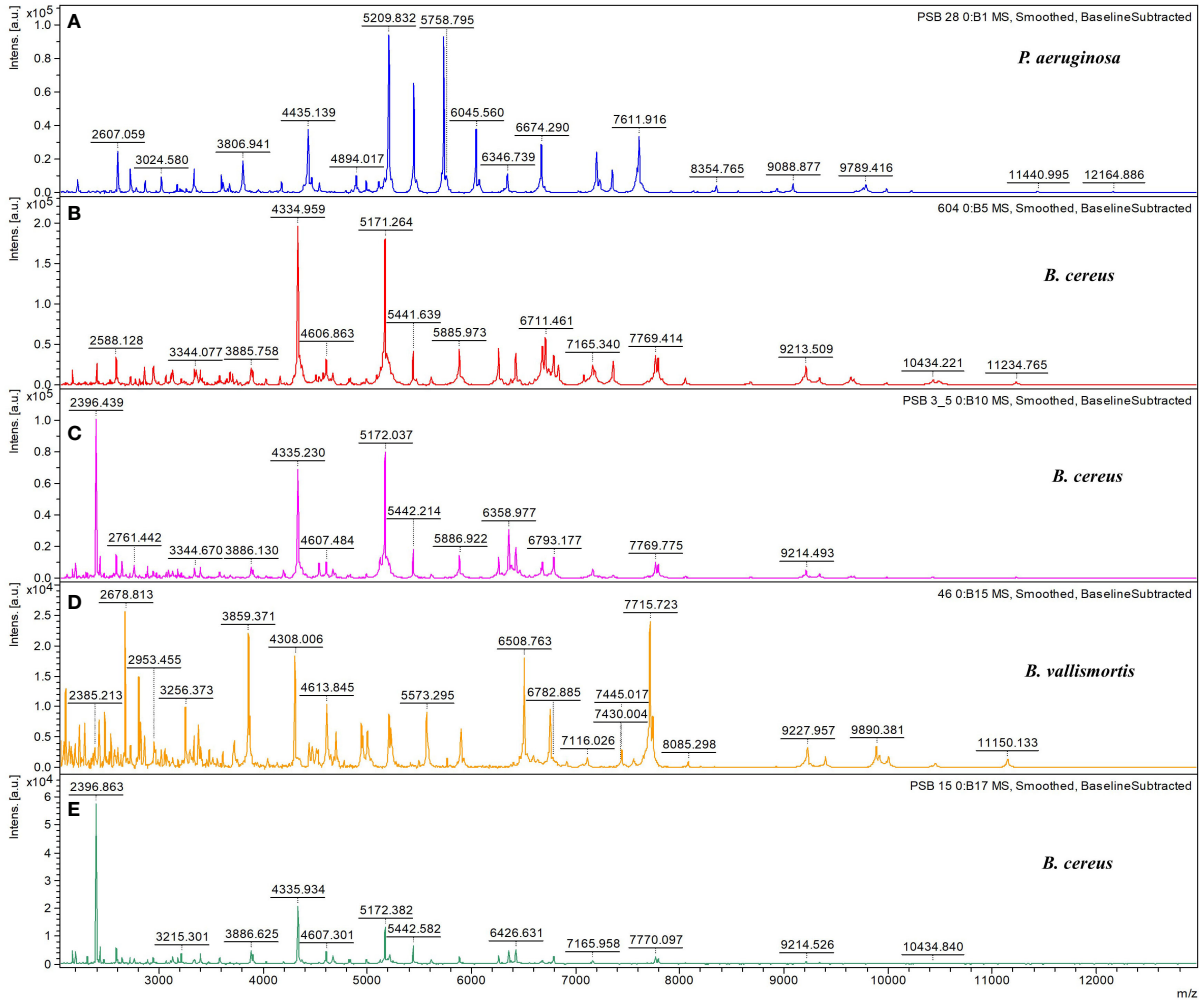
Characteristic MALDI–TOF/TOF MS spectra. Characteristic spectra of **(A)** *Pseudomonas aeruginosa*, **(B)** *Bacillus cereus*, **(C)** *B cereus*, **(D)** *B vallismortis*, and **(E)** *B cereus* generated using the Bruker BioTyper MALDI–TOF/TOF MS system. Absolute intensities of ions are shown on the y–axis, and the masses (*m*/*z*) of ions are shown on the x–axis. The *m*/*z* values represent the mass–to–charge ratios.

The spectra of five microbial strains analyzed with FlexAnalysis 3.4 and MALDI–BioTyper v.4.0 (Karlsruhe, Germany) were used to compare the mass fingerprints of samples with the library database. The analysis revealed that the microbial strains at the genus level presented log score values of >2.000, corresponding to species–level identification. Four species were identified as belonging to the genus *Bacillus*, and one was identified as *Pseudomonas aeruginosa* (**Table 1**). The characteristic spectra of the five microbial species are presented in **Figure 1**.

**Table 1:** Microbial species identified.

Samples	Microbial species	Score	Remark
PSB 28	*Pseudomonas aeruginosa*	2.435	*Pseudomonas aeruginosa* ATCC 27853 THL
BA604	*Bacillus cereus*	2.301	*Bacillus cereus* 4080 LBK
PSB 3–5	*Bacillus cereus*	2.100	*Bacillus cereus* 4080 LBK
TU–Orga21	*Bacillus vallismortis*	2.127	*Bacillus vallismortis* DSM 11031T DSM
PSB 15	*Bacillus cereus*	2.218	*Bacillus cereus* DSM 31T DSM

### 3.2 Inhibition of *M. oryzae* by microbial biocontrol agents

**Figure 2:**
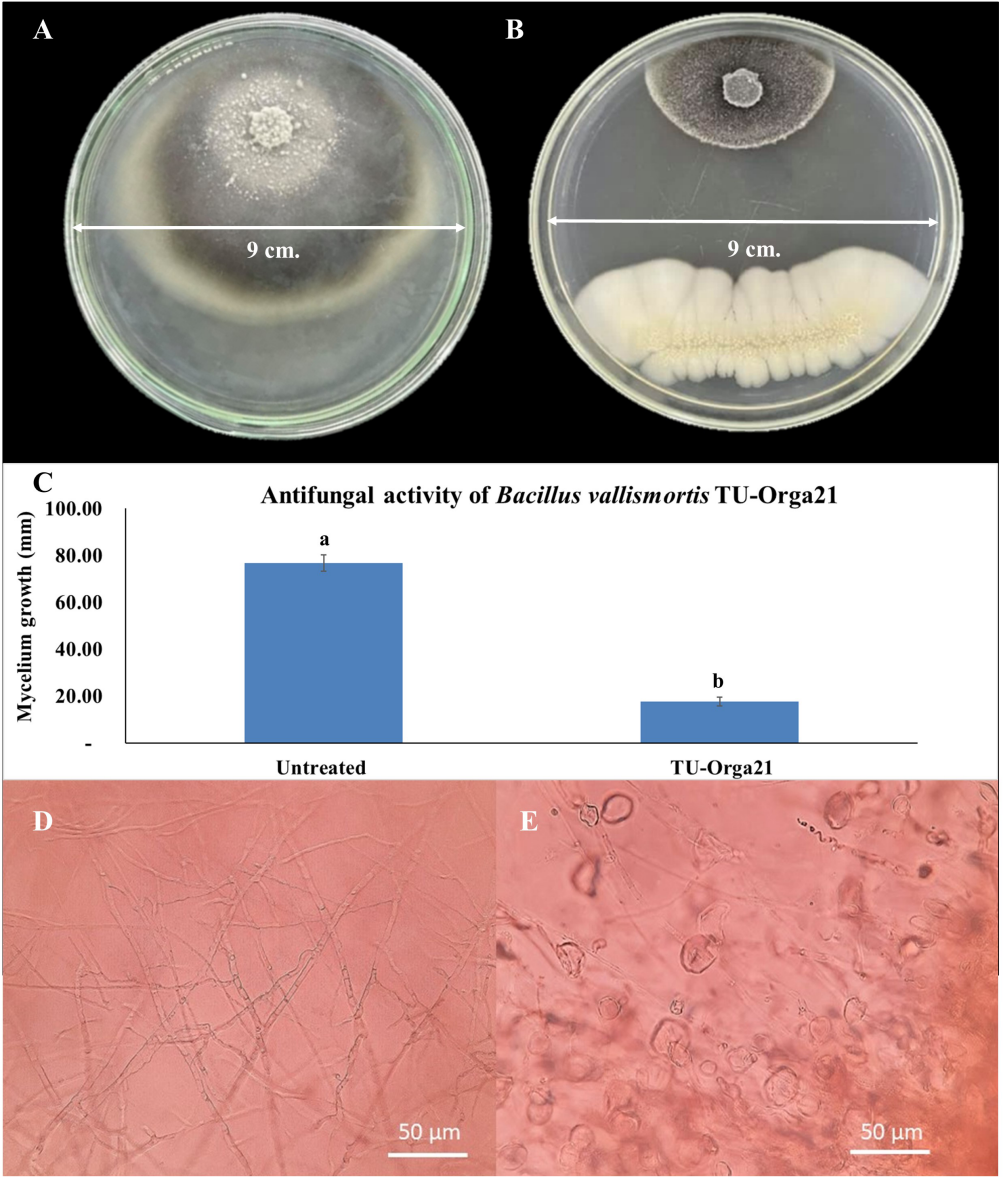
Observations of the antagonist testing against *Magnaporthe oryzae*. **(A)** *M. oryzae* mycelium growth in an untreated control plate. **(B)** Inhibition by *Bacillus vallismortis*. **(C)** *M. oryzae* mycelium growth bar plot in an untreated control plate compared with that in a *B vallismortis* –treated plate **(D)** Normal hyphae of *M. oryzae* in an untreated control plate. **(E)** Damaged hyphae of *M. oryzae* in a *B vallismortis* –treated plate. Means in the graph followed by different letters are significantly different according to DMRT at *p* = 0.01.

In the antagonist testing experiment, microbial biocontrol agents produced a strong effect against *M. oryzae* growth. Two *Bacillus* species showed comparable maximum percent inhibition of mycelial growth (**Table 2**). Specifically, *B. vallismortis* significantly suppressed *M. oryzae* growth by 77.03% (**Table 2**, **Figures 2A–C**). The hyphal morphology of *M. oryzae* was clearly affected by *B. vallismortis* under light microscopy. As such, the hyphae lost their normal growth and branching patterns, presenting wrinkled, twisted, and abnormal bending compared with hyphae in untreated samples (**Figures 2D, E**).

**Table 2:** Antagonistic activity of *Bacillus* sp. and *Pseudomonas* sp. against *Magnaporthe oryzae* causing rice blast, as assessed using the dual culture method.

Samples	Microbial species	Pathogen growth (mm)^1^	Inhibition (%)
PSB 28	*Pseudomonas aeruginosa*	70.55^d^	8.05
BA604	*Bacillus cereus*	45.00^c^	41.35
PSB 3–5	*Bacillus cereus*	72.65^d^	5.32
TU–Orga21	*Bacillus vallismortis*	17.625^a^	77.03
PSB 15	*Bacillus cereus*	71.47^d^	6.85
Positive control	Carbendazim	29.97^b^	60.93
Negative control	Untreated	76.72^d^	–

^1^ Each value represents the mean of four replicates. Means in the column followed by different letters (a, b, c, d) are significantly different according to DMRT at p = 0.05. -, no inhibition.

### 3.3 Effects of the TU–Orga21 biosurfactant on *M. oryzae* spore development

**Figure 3:**
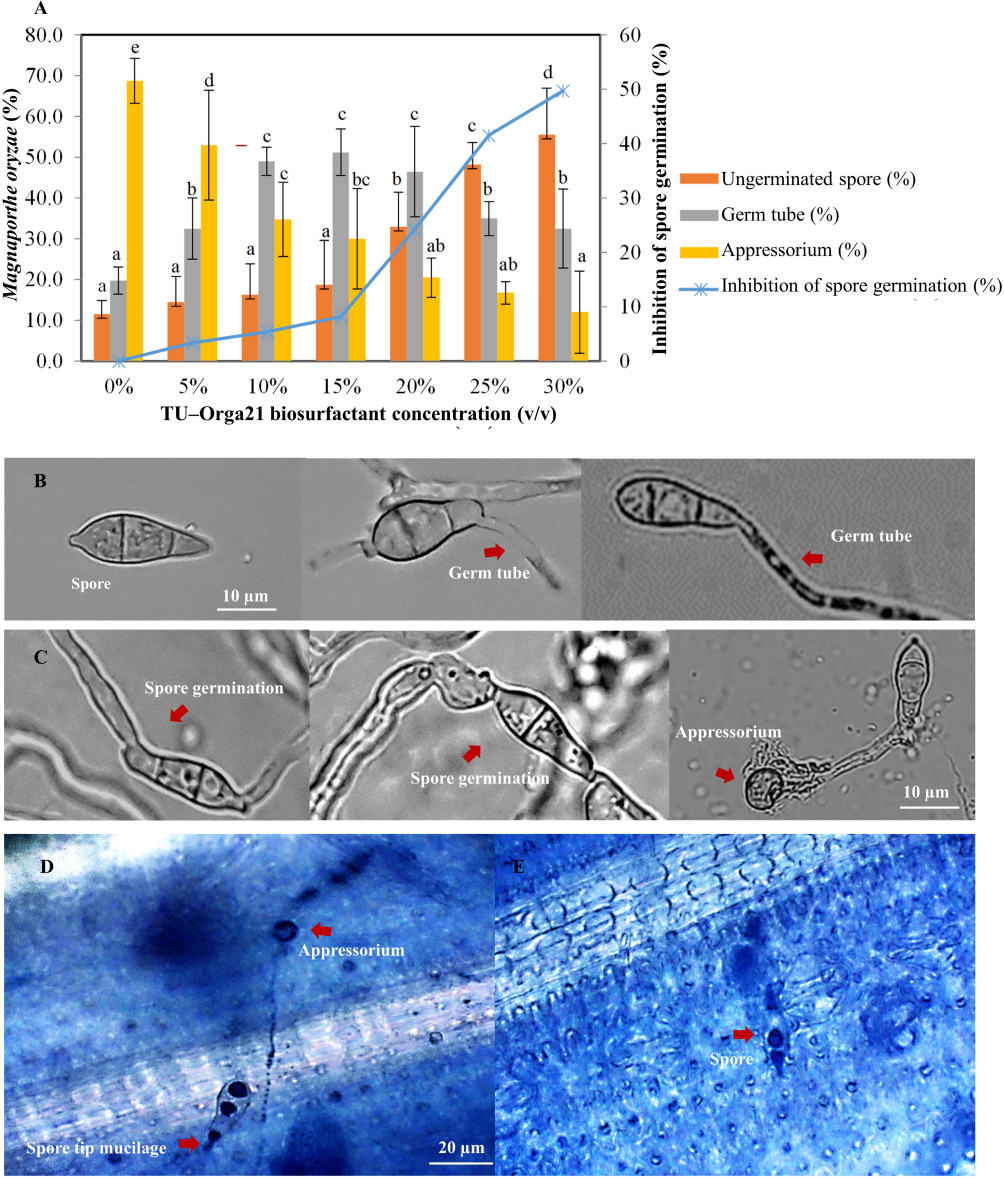
Efficacy of the *Bacillus vallismortis* biosurfactant. **(A)** Percentage of spore, germ tube, and appressorium (left ordinate) and inhibition of spore germination compared with the control treatment (0%) at 16 hpi (right ordinate). **(B)** Spore and spore which produced germ tube on hydrophobic coverslips in *B vallismortis* biosurfactant-treated **(C)** Spore germination and appressorium formation on hydrophobic coverslips in control. **(D)** Spore tip mucilage and appressorium formation in control sample. **(E)** Spores without germination in 30% *v*/*v* of biosurfactant-treated sample. Values are presented as the mean of four replicates. Means in the graph followed by different letters is a are significantly different according to DMRT at *p* = 0.05.

To determine the efficacy of the *B. vallismortis* biosurfactant against the primary infection process of *M. oryzae*, the percentage of spore, germ tube and appressoria formed on rice leaves at different concentrations were calculated as spore number of each development stage divided by the total number of spores. The percentages of germ tubes and appressoria formed in the control samples (0% *v*/*v* of biosurfactant) were 19.75% and 68.75%, respectively whereas spore without germination was 11.30%. Appressoria formation decreased with increasing biosurfactant concentration (53.00%, 34.75%, 30.00%, 20.50%, 16.75%, and 12.00% at 5%, 10%, 15%, 20%, 25%, and 30%, respectively). Meanwhile, germ tube formation was higher when using 15% *v*/*v* of biosurfactant (51.25%), although the difference was non–significant compared with values at 10% and 20% *v*/*v* biosurfactant doses (49% and 46.50%, respectively), whereas at 25% and 30% *v*/*v* biosurfactant doses (35.00% and 32.50%, respectively) were shown significantly lower. However, spore germination including germ tube and appressorium for each of the treatment was converted to percent inhibition compared with the control sample. The increase of biosurfactant concentration effectively increased the inhibition of spore germination by 50% compared with the control (**Figure 3**).

### 3.4 MALDI–TOF MS analysis of the TU–Orga21 biosurfactant

**Figure 4:**
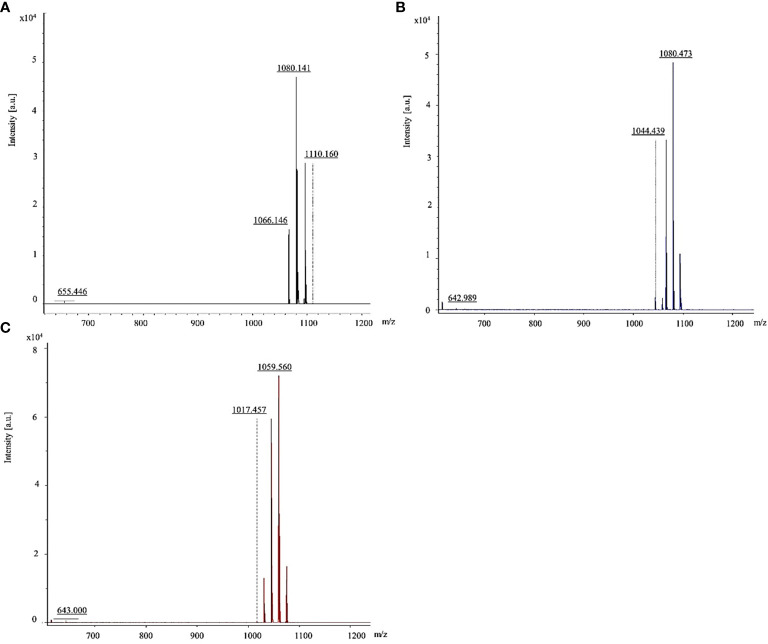
MALDI-TOF/TOF mass spectra of lipopeptides detected the range of 600–1,200 *m*/*z*. **(A)** Iturin A and surfactin in biosurfactant produced by *Bacillus vallismortis* assigned. **(B)** Iturin A standard. **(C)** Surfactin standard.

The *B. vallismortis* biosurfactant was further analyzed using MALDI–TOF/TOF MS, and intense signals were detected in the *m*/*z* range of 600–1,200. Results of molecular mass–ion analysis showed that the samples obtained from the *B. vallismortis* biosurfactant contained three primary peaks at 1,066, 1,080, and 1,110 *m*/*z* (**Figure 4A**). These groups of peaks corresponded to iturin A at 1,044, 1,080, and 1,100 *m*/*z* (**Figure 4B**) and surfactin at 1,017 and 1,059 *m*/*z* (**Figure 4C**) according to their standards. Although the peaks detected at 1,066 *m*/*z* did not exactly match with the established standard, these readings can be assigned as surfactin species, as they are detected in the range of *m/z* 1,000–1,074 (Vater et al., 2002).

### 3.5 Activity of defense-related intermediates induction by TU–Orga21 crude biosurfactant

**Figure 5:**
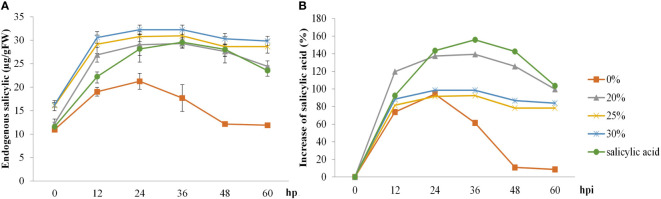
Endogenous salicylic acid (SA) activity in ‘KDML 105’ treated with biosurfactant following *Magnaporthe oryzae* infection. **(A)** Endogenous SA content and **(B)** relative increase in endogenous SA content.

The effects of the *B. vallismortis* biosurfactant on the endogenous SA content were evaluated at pre– and post–inoculation time points. Sprayed SA and biosurfactant doses on the rice plants increased endogenous SA content at 12, 24, and 36 hpi, but the values dropped at 48 hpi. In contrast, endogenous SA content in control samples (0% *v*/*v* biosurfactant) increased at 24 hpi and dropped at 36 hpi. Regarding relative increase, the highest endogenous SA content was recorded in SA treatment (160%), followed by that in the 20% *v*/*v* biosurfactant treatment (140%) (**Figure 5**).

**Figure 6:**
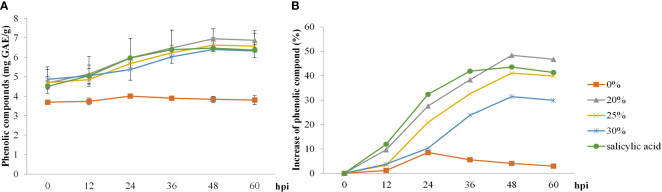
Total phenolic compound activity in ‘KDML 105’ treated with biosurfactant following *Magnaporthe oryzae* infection. **(A)** total phenolic compound content and **(B)** relative increase in total phenolic compound content.

Both biosurfactant and SA treatments elevated total phenolic content post–inoculation, with a faster increase from 12 to 48 hpi, whereas total phenolic content in the control treatment was only slightly increased at 24 hpi. The highest value of total phenolic content (48%) in rice leaves was recorded at 48 hpi following the 20% *v*/*v* biosurfactant treatment. On the contrary, phenolic content (8.5%) in the control treatment (0% *v*/*v* biosurfactant) showed lower percentage increase at 24 hpi (**Figure 6**).

**Figure 7:**
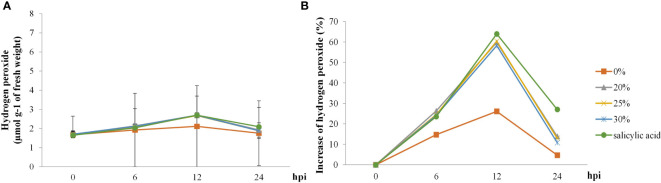
The hydrogen peroxide activity in ‘KDML 105’ treated with biosurfactant following *Magnaporthe oryzae* infection. **(A)** hydrogen peroxide content and **(B)** relative increase in hydrogen peroxide content.

H_2_O_2_ levels in rice plants started increasing at 6 hpi but decreased at 24 hpi in all treatments. H_2_O_2_ levels in rice plants treated with SA showed the highest increase (63%), followed by those in rice plants treated with the biosurfactant (~58%–60%) at 12 hpi; increase in the control treatment was only 25% (**Figure 7**).

### 3.6 Rice mesophyll biochemical change of elicitation-plant

**Figure 8:**
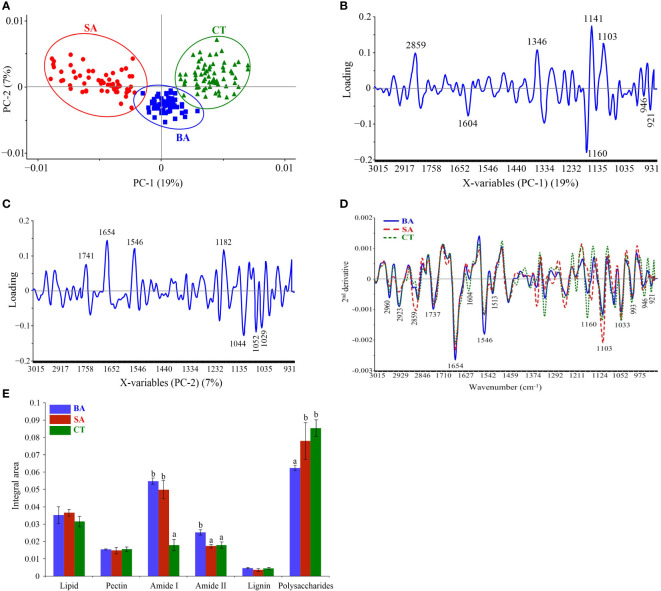
SR-FT-IR spectra of mesophyll in rice leaf tissue. **(A)** Principal component analysis (PCA). **(B)** Loading plots from PCA analysis (PC1). **(C)** Loading plots from PCA analysis (PC2). **(D)** Overlay of the average second derivative spectrum. **(E)** Integral areas of absorbance between 3,000 and 900 cm^-1^ Means in the graph followed by different letters are significantly different according to DMRT at *p* = 0.05. BA, *Bacillus vallismortis* –treated sample; SA, salicylic acid–treated sample; CT, control water–treated sample.

SR–FT–IR spectra were used for monitoring changes in rice biochemical composition corresponding to rice leaf mesophyll following treatment with the *B. vallismortis* surfactant compared with composition in the positive (SA) and negative (water–mock) control treatments. Three clearly distinguishable groups were noted: *B. vallismortis*, SA, and water–mock. Clusters of samples and PCA biplots were described by the first (PC1, 19%) and second (PC2, 7%) principal component (**Figure 8A**). PC1 showed high positive loadings at 2,859, 1,346, 1,141, and 1,103 cm^-1^, related to the positive score plots of SA–treated samples (positive controls). PC1 showed high negative loadings at 1,604, 1,160, 946, and 921 cm^-1^, related to the negative score plots of water–treated samples (negative controls) (**Figure 8B**). PC2 high positive loadings at 1,741, 1,654, 1,546, and 1,182 cm^-1^, related to the positive score plots of *B. vallismortis*–treated samples (**Figure 8C**). Spectral band variability through loading plots associated with the second derivative of the average spectra in the mesophyll showed different regions of the samples. The spectral region at 3,000–2,800 cm^-1^ from samples revealed peaks at 2,960, 2,923, and 2,859 cm^-1^, corresponding to the CH2 and CH3 parts of lipid groups. The region at 1,700–1,500 cm^-1^, corresponding to proteins and peptides, revealed peaks at 1,737, 1,654, 1,604, 1,546, and 1,513. The region at 1,300–900 cm^-1^, corresponding to polysaccharides and carbohydrates, revealed peaks at 1,160, 1,103, 1,033, 993, 946, and 921 cm^-1^ (**Figure 8D**). All these spectral averages were used to quantitatively identify the molecular structures of biochemical compounds and to obtain the integral areas of the biochemical segments of samples. From the integral area, the amounts of Amide I and Amide II were significantly increased but the amounts of polysaccharide were significantly decreased in *B. vallismortis* –treated samples compared with those in negative controls (*p*< 0.05) (**Figure 8E**).

### 3.7 Effect of the TU–Orga21 biosurfactant as an inducer of the *M. oryzae* infection process

**Figure 9:**
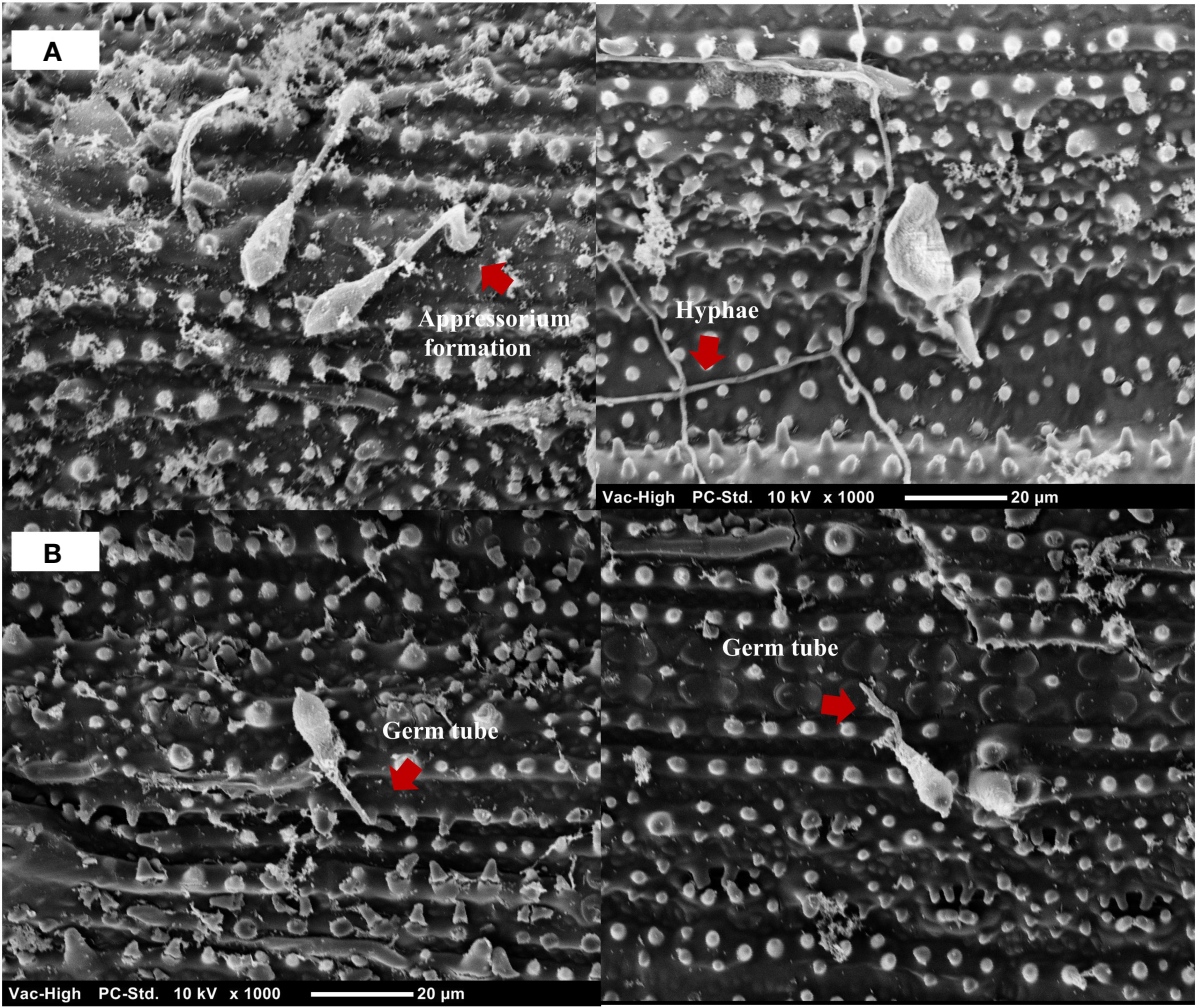
Scanning electron micrograph of *Magnaporthe oryzae* on detached rice leaves at 24 hpi. **(A)** *M. oryzae* successfully established appressoria and hyphae on the leaves of the untreated rice plant. **(B)** *M. oryzae* conidia adhesion on the leaves of *Bacillus vallismortis* –treated rice plant.

Observations of the infection process of *M. oryzae* at 24 hpi revealed that *M. oryzae* successfully infected and colonized untreated leaves, presenting appressorium formation and hyphal development. During the same period, treatment with the *B. vallismortis* biosurfactant as an inducer prevented the hyphal invasion in rice tissue by *M. oryzae* (**Figure 9**).

### 3.8 Reduction of rice blast disease severity

**Figure 10:**
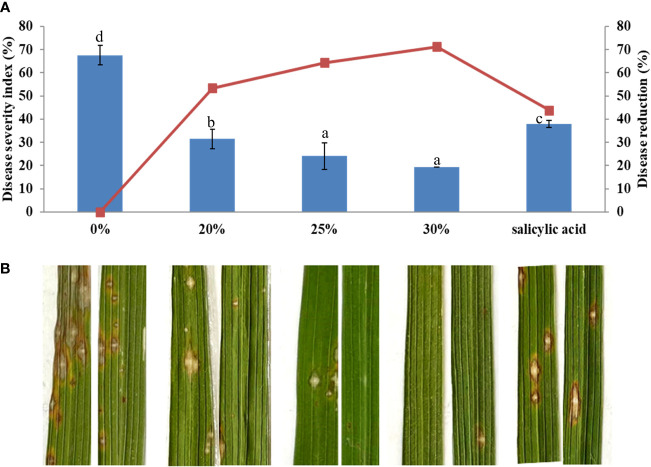
Effectiveness of the *Bacillus vallismortis* biosurfactant against rice blast caused by *Magnaporthe oryzae* in ‘KDML 105’. **(A)** Disease severity and disease reduction. **(B)** Visible symptoms on treated leaves at 21 days post-inoculation. Means in the graph followed by different letters are significantly different according to DMRT at p = 0.05.

The efficacy of the *B. vallismortis* biosurfactant at a range of concentrations, including 20%, 25%, and 30% *v*/*v*, against rice blast was evaluated under greenhouse conditions. SA as the inducer was used as the positive control and 0% *v*/*v* biosurfactant as the negative control. Biosurfactant treatment before inoculation reduced disease severity compared with both positive and negative control treatments, as expressed by higher disease reduction percentages. The initial disease symptoms appeared at 14 dpi. Common rice blast symptoms were detected as elliptical lesions or white–grey spots surrounded by necrotic brown edges. At 21 dpi, disease severity was respectively 67.59% and 37.96% in negative and positive control samples. Treatment with 30% *v*/*v* biosurfactant showed the lowest disease severity (19.44%), followed by treatments with 25% and 20% *v*/*v* biosurfactant (24.07% and 31.48%, respectively); these values were significantly lower than that in the positive control treatment (37.96%) (**Figure 10**).

## 4 Discussion

Beneficial microorganisms have been considerably characterized for their antagonism against common agricultural pathogens. Moreover, such microorganisms have been exploited as eco–friendly substitutes for pesticides, as they produce antimicrobial compounds and activate plant immunity to combat a broad range of plant pathogens. Most studies addressing beneficial microorganisms have explored their antimicrobial activities or plant defense induction potential. Therefore, in the present study, we analyzed the control ability of five representative microorganisms isolates from paddy fields near the rhizospheric region against rice blast through both direct and indirect antagonism.

*B. vallismortis* exhibited potent antagonistic activity against *M. oryzae* (>50%), as determined based on the production of antimicrobial substances. Furthermore, we investigated the ability of the *B. vallismortis* biosurfactant to inhibit *M. oryzae* development and observed that at high concentrations, the biosurfactant can effectively suppress spore germination and appressorium development in *M. oryzae*. These findings are consistent with previous reports that lipopeptide biosurfactants from *B. subtilis* inhibited the germination of *M. oryzae*—the causal agent of wheat blast (Chakraborty et al., 2020). In previous studies, different *Bacillus* strains, including *B. subtilis*, *B. cereus*, *B. amyloliquefaciens*, *B. licheniformis*, and *B. pumilus*, have been applied as biocontrol agents against rice blast for organic and sustainable farming. For instance, *B. vallismortis* R2 could be used as an alternative to combat several plant pathogenic fungi, including *Alternaria* sp., *Rhizoctonia oryzae*, *Fusarium* sp., *Colletotrichum* sp., *Helminthosporium* sp., and *Magnaporthe grisea* (Kaur et al., 2015; Sha et al., 2020). In addition, *B. vallismortis*, which produce numerous antimicrobial bioactive metabolites, such as thermostable alkalophilic cellulose, bacillomycin D, surfactins, iturins, and fengycins, has been tested for its biocontrol properties (Zhao et al., 2010; Kaur et al., 2017).

Our MALDI–TOF/TOF MS analysis of the *B. vallismortis* biosurfactant revealed the production of two major classes of lipopeptide antibiotics based on secondary metabolites, including iturin A and surfactin. The groups of peaks at *m/z* values between 1,000 and 1,074 correspond to surfactins and those between 1,076 and 1,150 correspond to iturins (Vater et al., 2002). Iturin A peak in the sample was detected at 1,080 *m/z*, consistent with its standard (1,080 *m/z*). Surfactin peak in the sample was detected at 1,066 *m/z*, close to its standard (1,059 *m/z*). The surfactin peak was different from its standard, although it was still in the range of surfactin species (Vater et al., 2002; Hoefler et al., 2012).

Iturins have been identified as antifungal substances that degrade the cell wall, inhibit substance exchange and metabolic processes, and lower viscoelastic fluid pressure in fungal cells (Jiang et al., 2020). The antifungal mechanism of iturin lipopeptides is associated with their interaction in the cytoplasmic membrane, which increases potassium permeability (Maget-Dana and Peypoux, 1994). Furthermore, surfactins destroy the internal structure of cells through digesting the cytoplasm and organelles (Xiao et al., 2021). Iturins and surfactins not only exhibit a strong antifungal activity but also act as bioactive molecules to induce plant defense (Raaijmakers et al., 2010). Several studies have reported that lipopeptides, in addition to iturins, surfactins, and fengycins, activate plant ISR, through which the gene expression of defense proteins, including phenylalanine lyase, peroxidases peroxidases, and other pathogenesis–related (PR) proteins, is promoted. This mechanism is essentially responsible for the innate immunization to inhibit pathogens in different plants. Previous studies have demonstrated that lipopeptides, as products of bacterial biosurfactants, activate specific plant defense pathways against pathogens, such as pathways against *Colletotrichum gloeosporioides* in strawberry, pathways against *Sclerotinia sclerotiorum* in tomato, and pathways against *M. oryzae* in rice (Yamamoto et al., 2015; Farzand et al., 2019; Lam et al., 2021).

The pivotal roles of the *B. vallismortis* biosurfactant in plant immune interactions was further investigated in the present study by analyzing the changes in host defense intermediate biochemicals related to enhanced resistance against *M. oryzae*. Spraying the *B. vallismortis* biosurfactant three times before pathogen inoculation resulted in the establishment of plant immunological parameters, including increases in endogenous SA and phenolic levels (*p*< 0.05).

SA, jasmonic acid (JA), and ethylene (ET) are important hormonal signal molecules involved in biotic stress responses during plant–pathogen interactions. Typically, SA–mediated defense responses play a central role in local and systemic–acquired resistance against biotrophic and hemi–biotrophic pathogens, whereas ET/JA–mediated responses contribute to defense against necrotrophic pathogens (Bari and Jones, 2009; D. Tamaoki et al., 2013). *M. oryzae* is a hemi–biotrophic fungus that causes rice blast; it assumes an initial biotrophic stage during which the plant immune system is suppressed, followed by the necrotrophic stage that promotes plant cell death (Fernandez and Orth, 2018).

The SA pathway is the dominant signaling cascade under pathogen attacks due to the presence of communication channels that can promote generation of energy and synthesis of potential intermediates and enzymes to inhibit pathogens (Koo et al., 2020). Studies on defense responses of multiple plant species, including rice, soybean, and tobacco, have reported that pathogen infection leads to SA accumulation (Yalpani et al., 1993; Bawa et al., 2019; Liang et al., 2022). At the early stages of pathogen infection, plants recognize and activate the defense signals as well as induce damage–associated molecular patterns (DAMPs), including extracellular protein fragments, peptides, nucleotides, and amino acids (Hou et al., 2019). The transcription factors for *PR* genes activate SA to mediate host response in infected plant cells (Hou et al., 2019). Kawagoe et al. (2015) characterized the signaling pathways of ISR stimulated by lipopeptides in *Arabidopsis* through the SA and JA signaling pathways, which activated the transcription of defense genes (Kawagoe et al., 2015). Moreover, SA accumulated around the infected area generates HRs at the initial infection site (Daisuke Wildermuth et al., 2001; Tamaoki et al., 2013). Lakkis et al. (2019) reported that *Pseudomonas fluorescens* is an inducer of ISR in grapevine, which activates both immune response and priming phenomenon under *Botrytis cinerea* infection; this event is related to the constitutively enhanced expression of SA–related genes and increased total phenolic content (Lakkis et al., 2019).

The involvement of phenolic compounds in the defense reaction is a crucial physiological function in several plants, such as lettuce, banana, and rice plants, to offer resistance against multiple biotic stressors (Sedlářová and Lebeda, 2001; Dallagnol et al., 2011; Fortunato et al., 2012; Pratyusha, 2022). According to our results, the total phenolic content showed the highest increase in the 20% *v*/*v* biosurfactant treatment. In contrast, in the 30% *v*/*v* biosurfactant treatment, the magnitude of increase was lower. This may be because *B. vallismortis* produces iturin A and surfactin in the biosurfactant, which produce a direct antimicrobial effect against the pathogen; thus, the inoculum should have a lower density than in the other treatments. Similarly, H_2_O_2_ did not produce the strongest effect as it is a defense intermediary contributing to program cell death (PCD) under pathogen attack. These observations are consistent with previous reports that phenolic production is linked to SA–induced resistance responses, which are often augmented after a pathogen attack leading to HR and contributing to subsequent cell death (Wallis and Galarneau, 2020). Betsuyaku et al. (2018) reported higher levels of SA in concentric patterns of cellular responses in effector–triggered immunity. PAMP–triggered immunity induces an HR and localized PCD to inhibit pathogen spread, which effectively protects the plant tissues from disease severity (Betsuyaku et al., 2018; Radojičić et al., 2018). In the present study, the comparison of H_2_O_2_–mediated PCD and initiation of defense responses against *M. oryzae* infection between treated and untreated samples revealed significant temporal differences in the accumulation of H_2_O_2_. This result confirms that in the present experiment, plant defense activity exclusively occurred after *M. oryzae* infection. The higher H_2_O_2_ activity suggests that reactive oxygen species (ROS) contribute to the disruption of the electron transport chain at the early stages of infection (6–12 hpi) produced by elicitation with biosurfactant. The higher H_2_O_2_ levels may enhance the ability of cells to detoxify H_2_O_2_, which may generate damage–related signals and activate transcriptional regulators of PCD–related genes. PCD under host–pathogen interactions in the elicitation affects the rapid inhibition of pathogen development. These results are consistent with the plant timing defense initiation theory and corroborate the observations of Ye et al. (2021), who reported increased ROS concentrations after pathogen infection, leading to a change in the antioxidant status of cells and induction of plant responses in terms of immunity–related cell death (Ye et al., 2021).

**Figure 11:**
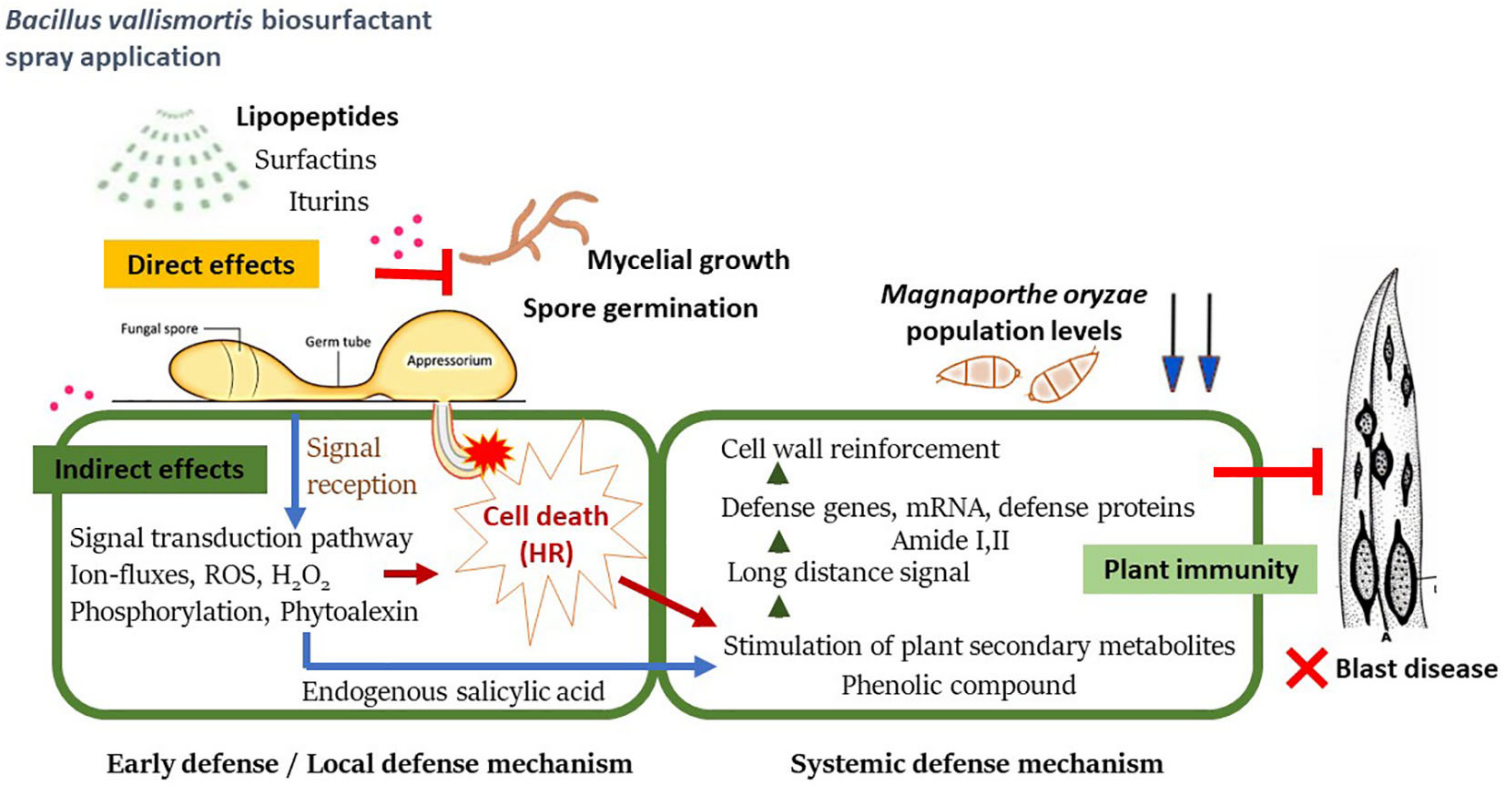
A proposed schematic model of systemic resistance in rice plant against *Magnaporthe oryzae* after being treated with *Bacillus vallismortis* biosurfactant.

This indirect inhibitory effect of the biosurfactant causes a shift in intracellular biochemical elements, which were detected using SR–FT–IR microspectroscopy. The biomolecule–related intensities from average spectra revealed significantly higher agglomeration of Amide I (1,600–1,800 cm^-1^) and Amide II (1,470–1,570 cm^-1^) proteins. A plant protein peptide bond is an amide group as well as a type of endogenous factor in disease resistance to encourage plant–microbe interaction (Liu et al., 2022). Plant peptides induce immune responses that are established at the receptor level of plant peptides in response to pathogen invasion, including the induction of defense–related phytoalexin, lignification, and PR proteins (Hu et al., 2018; Kumar et al., 2018). Moreover, significantly less polysaccharides (1,200–900 cm^-1^), such as carbohydrates and sugar, were accumulated in biosurfactant–treated samples. This may be because during the infection process, plants adapt or alter their energy storage to trigger defense responses against pathogens (Tauzin and Giardina, 2014). We further examined the *M. oryzae* infection process on leaves using SEM. Elicitation of rice plants with the biosurfactant disrupted the development process of *M. oryzae*, confirming that the biosurfactant produced an indirect inhibitory effect on host plant infection (**Figure 11**).

## 5 Conclusions

The results of the present study confirmed that the *B. vallismortis* biosurfactant exhibits antibacterial activity against *M. oryzae*–the causative agent of rice blast–through both direct and indirect inhibitory effects. Moreover, experimental data confirmed that the biosurfactant, as an elicitor, effectively reduced rice blast severity through the production of antibiotic substances, including iturin A and surfactin. In addition, levels of endogenous SA, phenolics, H_2_O_2_, amide I and, amide II, all of which are defense intermediaries, were increased, contributing to against *M. oryzae* during pathogenesis.

## Data availability statement

The original contributions presented in the study are included in the article/supplementary material. Further inquiries can be directed to the corresponding author.

## Author contributions

WT and DA: Conceptualization and design. WT, AS, SS, and SN: Implementation, investigation, and analyzed the data. WT and SS: Arranged the results, discussion, and writing the original draft. WT, SS, and DA: Revised the manuscript. All authors contributed to the article and approved the submitted version.
